# Propensity Score-matched Comparison of WEB 17 and WEB 21 with 4–7 mm Device Sizes for the Treatment of Unruptured Intracranial Aneurysms

**DOI:** 10.1007/s00062-024-01430-2

**Published:** 2024-06-19

**Authors:** Lukas Goertz, Thomas Liebig, Eberhard Siebert, David Zopfs, Lenhard Pennig, Marc Schlamann, Alexandra Radomi, Franziska Dorn, Christoph Kabbasch

**Affiliations:** 1 Department of Radiology and Neuroradiology, University of Cologne, Faculty of Medicine and University Hospital, Kerpener Straße 62, 50937 Cologne, Germany; 2 Department of Neuroradiology, University Hospital Munich (LMU), Marchioninistraße 15, 81377 Munich, Germany; 3 Department of Neuroradiology, University Hospital of Berlin (Charité), Charitéplatz 1, 10118 Berlin, Germany; 4 Department of Neuroradiology, University Hospital of Bonn, Venusberg-Campus 1, 53127 Bonn, Germany

**Keywords:** Endovascular, Flow-disruption, Intracranial aneurysm, Intrasaccular

## Abstract

**Purpose:**

The WEB 17 system represents the fifth generation of Woven Endobridge (WEB) flow disruptors and features a low profile with fewer wires than its predecessor, the WEB 21. The present study compares the safety and efficacy of the WEB 17 and WEB 21 for the treatment of unruptured cerebral aneurysms with 4–7 mm device sizes, which were available for both systems.

**Methods:**

Patient and aneurysm characteristics, complications, clinical outcome and angiographic results were retrospectively analysed. 1:1 propensity score matching was performed to adjust for minor baseline differences between the groups.

**Results:**

Sixty aneurysms treated with WEB 21 and 90 with WEB 17 were included. The overall failure rate (deployment failure and adjunctive stent) was significantly higher with WEB 21 (16.7%) than with WEB 17 (3.3%, *p* < 0.01). The rates of neurological events between WEB 21 (6.7%) and WEB 17 treatment (1.1%) were not significantly different (*p* = 0.08). Also, procedural morbidity was comparably low in both groups (WEB 21: 3.3%, WEB 17: 0%, *p* = 0.16). The rates of complete/adequate occlusion at follow up were 69.7%/86.4% for WEB 17 vs. 80.4%/91.3% for WEB 21 at short-term (*p* = 0.27), and 64.5%/83.9% vs. 75.9%/86.2% at mid-term (*p* = 0.41), respectively. Propensity score matching confirmed the results of the unmatched series.

**Conclusion:**

WEB 17 and WEB 21 had a similar safety and efficacy profile, but WEB 17 was associated with an improved feasibility. Prospective studies with long-term follow-up will define the full potential of the WEB 17 system.

## Introduction

The Woven Endobridge (WEB; Microvention, Aliso Viejo, CA, USA) was first to show that intrasaccular flow-disruption is an effective treatment modality for a wide variety of intracranial aneurysms, including wide-necked bifurcation aneurysms [1, 12, 13]. Since its introduction in 2010, technological advances and increased expertise have broadened the range of conditions amenable to WEB treatment [2]. Of note, the evolution of the WEB design from a dual-layer configuration (WEB-DL) to a single-layer design (WEB-SL), and alterations of the marker and wiring allowed the use of a smaller delivery catheter [3]. The introduction of the WEB 17 device has refined the delivery process, allowing positioning through a 0.017″ microcatheter for implants up to 7 mm in size [4]. The WEB 17 series includes additional sizes, such as a small device (width = 3 mm), half mm step sizes (widths = 3.5 and 4.5 mm), and flat devices with a height of only 2 mm.

This miniaturization is accompanied by a reduction in both the number of wires (from 144–216 wires in WEB 21 to 72–108 wires in WEB 17) and wire diameter. It is thus essential to ensure that WEB 17 maintains the same level of safety and efficacy as its predecessor. While several case studies, including the prospective CLEVER (Clinical EValuation of WEB 17 device in Intracranial AneuRysms) study, have suggested a favorable safety and efficacy profile for WEB 17 that may exceed that of WEB 21, there are only few comparative studies of these devices [4, 5].

The present study compares the feasibility, safety and efficacy of the WEB 17 and WEB 21 for the treatment of unruptured cerebral aneurysms with 4–7 mm device sizes, as these sizes were available for both systems. In addition, 1:1 propensity score matching was performed to address potential confounding due to differences in baseline characteristics.

## Methods

This is a multicenter, retrospective, observational study of individuals treated with the WEB device between June 2015 and September 2023. Ethical approval for the collection of anonymized data was obtained from the respective local ethics committees associated with each participating center. Institutional policy specifically exempted the need for individual approval in the context of this retrospective, observational study.

### Inclusion Criteria

Consecutive patients treated with the WEB device during the designated study period underwent a comprehensive review based on an intention-to-treat framework. Inclusion criteria included cases that met the following conditions: (1) either successful or attempted intervention using a WEB SL or SL-sphere (SLS) with a device width of 4–7 mm, respectively, (2) additional stent placement if performed as a salvage procedure, (3) aneurysm dome width 3.0–6.5 mm, (4) any aneurysm height, and (5) unruptured/naïve and recurrent aneurysm status. Exclusion criteria included: (1) treatment involving the use of the WEB DL, (2) partial intrasaccular thrombosis, (3) mycotic aneurysms, (4) fusiform aneurysms, (5) ruptured aneurysms, (6) aneurysms associated with arteriovenous malformations, (7) procedures with additional coiling and (8) cases without available data on WEB type/size. The included range of aneurysm widths (3.0–6.5 mm) was chosen because these aneurysm sizes can be treated with either WEB 21 or WEB 17 with device widths of 4–7 mm, as recommended by the manufacturer according to the device sizing charts [6, 7]. Ruptured aneurysms were excluded to homogenize the groups, as emergency cases were not performed under antiplatelet medication and the assessment of clinically relevant symptoms is difficult in intubated patients.

### WEB Treatment

WEB treatment procedures were performed using a Philips (Best, The Netherlands) and Siemens (Erlangen, Germany) biplane angiosuite. The dedicated VIA microcatheter from Microvention (Aliso Viejo, CA, USA) was predominantly used for WEB delivery, using a 0.017″ microcatheter for WEB 17 and a 0.021″ microcatheter for WEB 21. Since September 2020, aneurysms with a maximum diameter not exceeding 7 mm have been treated exclusively with the WEB 17. Implant sizes were selected using the manufacturer’s sizing chart, which accounts for aneurysm width and height.

The primary goal was to use the WEB device as the sole modality to treat all included aneurysms. However, the potential use of adjunctive stents or coils was considered a salvage option at the discretion of the neurointerventionalist. In general, stent placement was used to prevent WEB protrusion in aneurysms characterized by unfavorable anatomic features.

### Anti-aggregation Therapy

Antiplatelet therapy, consisting of a daily dose of 100 mg of acetylsalicylic acid (ASA), was initiated 5–7 days before the procedure and continued for 4–6 weeks. Antiplatelet therapy was then discontinued. A single bolus of 5000 IU heparin was administered immediately after inguinal puncture, accompanied by a continuous infusion of 1000 IU/hour aliquots throughout the procedure. In cases where additional stents were used as a rescue strategy, an intravenous infusion of body weight-adjusted tirofiban was initiated prior to stent placement and maintained for 16–24 h. This was followed by sequential loading with clopidogrel at a dose of 300 mg. After the procedure, a regimen of ASA 100 mg and clopidogrel 75 mg was administered for at least 4 months. Subsequently, ASA monotherapy was continued.

### Data Collection and Angiographic Evaluation

Patient demographics, procedural details, and procedural complications were determined by chart review. Procedural details included factors such as WEB size, WEB type (SL or SLS), delivery system specifications (WEB 17 or 21), fluoroscopy time, use of stents, and any instances of potential retreatment. Treatment failure was defined as the inability to adequately position the WEB within the aneurysm sac, necessitating subsequent removal and prompting alternative endovascular treatment or aneurysm clipping and as need for adjunctive stent placement as bailout option.

Procedural complications were comprehensively documented, including clinically relevant (symptomatic) complications and technical complications without clinical consequences. Neurological complications leading to deficits were classified as major if symptoms persisted for at least 7 days, whereas minor complications were characterized by resolution of symptoms within 7 days. Clinical outcomes were assessed at the time of hospital discharge using the modified Rankin Scale (mRS). Procedural morbidity was defined as a 1-point increase at discharge compared to baseline.

Conventional four-vessel digital subtraction angiography (DSA) was performed to determine number and locations of aneurysms. Aneurysm size parameters such as dome width (D), height (H), and neck width (N) were measured using 3D rotational angiography. These measurements were used to calculate the dome-to-neck ratio (D/N) and the aspect ratio (H/N). Aneurysms characterized by neck width ≥ 4 mm and/or D/N ratio ≤ 2 were classified as wide-necked.

The assessment of aneurysm occlusion was performed using the Raymond-Roy Occlusion Classification (RROC) scale, with complete occlusion and residual neck being considered as adequate occlusion. Follow-up was stratified into short-term (≤ 12 months) and mid-term (> 12 months) intervals for analysis.

### Statistical Analysis

Qualitative variables are expressed as numbers and percentages, while quantitative variables are presented as means with standard deviations. Statistical comparisons for qualitative variables are made using the chi-squared test and Fisher’s exact test, while quantitative variables were assessed using the Student’s t‑test for normally distributed data and the Wilcoxon-Mann-Whitney test for non-normally distributed data. The Shapiro-Wilk test was used to assess normality.

Individual propensity scores (PS) were calculated using a multivariate logistic regression model with WEB 17 treatment as the response variable. Covariates included in the model were patient age, aneurysm location, naïve/recurrent aneurysm status, aneurysm size, D/N ratio, and neck width. These individual propensity scores were then used to perform 1:1 matching, resulting in two comparable study groups. Statistical analyses were performed using SPSS software (IBM SPSS Statistics for Windows, version 25.0, Armonk, NY, USA), and a significance level of *p* < 0.05 was considered statistically significant.

## Results

### Patient and Aneurysm Characteristics

**Fig. 1:**
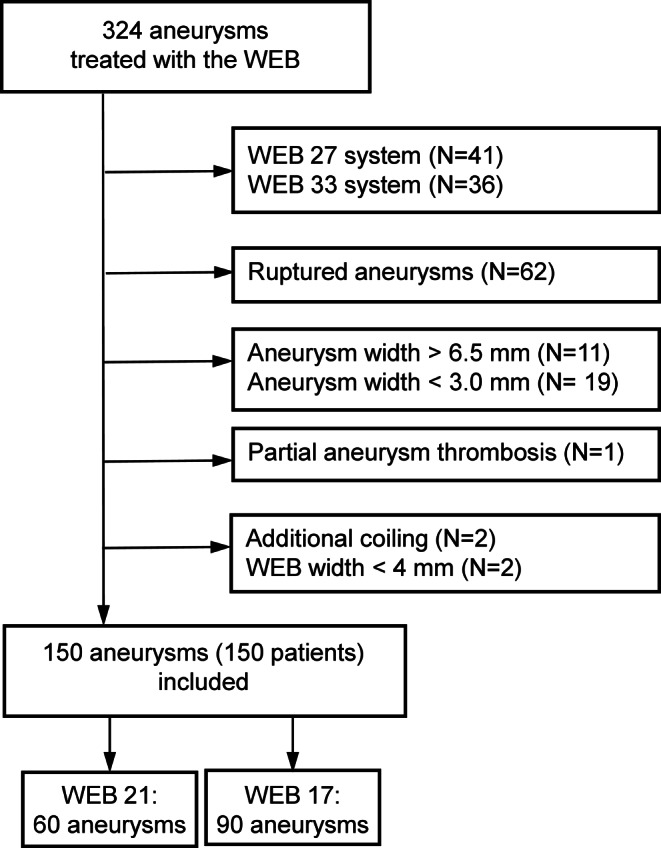
Flow chart of patient selection

The final study population consisted of 150 patients who underwent 150 WEB procedures (60 WEB 21, 90 WEB 17) for the treatment of 150 aneurysms. Patient selection is shown in Fig. 1. The mean patient age was 59.8 ± 10.9 years and 111 (74.0%) were female. Eleven (7.3%) aneurysms were recurrent, previously treated aneurysms. Aneurysms were located in the anterior circulation in 109 (72.7%) and in the posterior circulation in 41 (27.3%) cases, with 103 (68.7%) aneurysms being bifurcation aneurysms. The most common aneurysm locations were the anterior communicating artery (Acom) in 42 cases (28.0%) and the tip of the basilar artery (BA) in 33 cases (22.0%). The mean dome width was 5.0 ± 1.1 mm, the aneurysm height was 5.8 ± 2.2 mm, and the neck width was 3.6 ± 1.0 with a dome to neck ratio of 1.5 ± 0.4 and an aspect ratio of 1.7 ± 0.6. Patients treated with WEB 17 were significantly older (*p* < 0.01) and had aneurysms with smaller neck width (*p* = 0.02) than patients in the WEB 21 group. Baseline characteristics of both groups are shown in Table 1. All 60 WEB 21 cases were matched to 60 WEB 17 cases by 1:1 propensity score matching with no significant differences in baseline patient and aneurysm characteristics (Table 1).

**Table 1:** Baseline patient and aneurysm characteristics.

Parameter	Unmatched series			Propensity score-matched WEB 17 cases	
	WEB 21 (*N* = 60)	WEB 17 (*N* = 90)	*P*	WEB 17 (*N* = 60)	*P*
Patient age	56.3 ± 11.4	62.2 ± 9.9	< 0.01	59.8 ± 9.8	0.21
Female sex	46 (76.7%)	65 (72.2%)	0.54	44 (73.3%)	0.67
Recurrent aneurysms	5 (8.3%)	6 (6.7%)	0.70	3 (5.0%)	0.72
*Aneurysm location*	*–*		*0.33*	*–*	*0.55*
Anterior circulation	41 (68.3%)	68 (75.6%)	–	44 (73.3%)	–
Anterior communicating artery	11 (18.3%)	31 (34.4%)		23 (38.3%)	
Pericallosal artery	1 (1.7%)	4 (4.4%)		2 (3.3%)	
Middle cerebral artery bifurcation	12 (20.0%)	10 (11.1%)		5 (8.3%)	
M1-segment	0 (0%)	2 (2.2%)		1 (1.7%)	
Internal carotid artery	–			–	
terminus	4 (6.7%)	2 (2.2%)		1 (1.7%)	
paraophthalmic	8 (13.3%)	4 (4.4%)		3 (5.0%)	
Posterior communicating segment	5 (8.3%)	15 (16.7%)		9 (15.0%)	
Posterior circulation	19 (31.7%)	22 (24.4%)		16 (26.7%)	
Basilar artery tip	16 (26.7%)	17 (18.9%)		12 (20.0%)	
Vertebral artery	1 (1.7%)	0 (0%)		0 (0%)	
Superior cerebellar artery	0 (0%)	2 (2.2%)		1 (1.7%)	
Posterior inferior cerebellar artery	2 (3.3%)	3 (3.3%)		3 (5.0%)	
Bifurcation location	43 (71.7%)	60 (66.7%)	0.52	41 (68.3%)	0.69
*Aneurysm size*					
Dome width (mm)	5.1 ± 1.2	4.9 ± 1.1	0.21	5.0 ± 1.1	0.51
Height (mm)	6.0 ± 1.9	5.7 ± 2.4	0.46	5.8 ± 2.1	0.67
Neck width (mm)	3.8 ± 1.0	3.4 ± 1.0	0.02	3.6 ± 1.0	0.36
Dome-to-neck ratio	1.4 ± 0.3	1.6 ± 0.6	0.08	1.5 ± 0.4	0.38
Aspect ratio	1.6 ± 0.6	1.7 ± 0.6	0.50	1.7 ± 0.6	0.37
Wide neck	56 (93.3%)	87 (96.7%)	0.44	58 (96.7%)	0.68

mm = millimeter

### Procedural Details

Table 2 summarizes the procedural characteristics for both the matched and unmatched cohorts. WEB 21 deployment failed in 4 (6.7%) aneurysms (2 Acom, 2 paraophthalmic internal carotid artery [ICA]) compared to 2 (2.2%) WEB 17 cases (1 Acom, 1 BA tip), the difference was not significant (*p* = 0.22). Reasons for WEB failure were a sharp aneurysm-vessel angle in 3 cases, WEB protrusion in 1, inappropriate WEB size in 1, and a technical defect of detachment in 1. These cases were subsequently treated with stent-assisted coiling in 4 cases, balloon-assisted coiling in 1 and stand-alone coiling in 1.

**Table 2:** Procedural characteristics

Parameter	Unmatched series			Propensity score-matched WEB 17 cases	
	WEB 21 (*N* = 60)	WEB 17 (*N* = 90)	*P*	WEB 17 (*N* = 60)	*P*
Treatment failure overall	10 (16.7%)	3 (3.3%)	< 0.01	2 (3.4%)	0.03
WEB deployment failure	4 (6.7%)	2 (2.2%)	0.22	1 (1.7%)	0.68
WEB + adjunctive stents	6 (10.0%)	1 (1.1%)	0.03	1 (1.7%)	0.06
*WEB type*		*–*	0.28	–	0.05
Single-layer (SL)	45 (75.0%)	60 (66.7%)	–	35 (58.3%)	–
Single-layer sphere (SLS)	15 (25.0%)	30 (33.3%)		25 (41.7%)	
WEB width (mm)	5.8 ± 1.1	5.5 ± 1.1	0.09	5.5 ± 1.1	0.20
WEB/dome ratio	1.14 ± 0.14	1.14 ± 0.13	0.90	1.12 ± 0.13	0.54
Fluoroscopy time (min)	24.6 ± 19.1	24.0 ± 18.8	0.86	23.6 ± 19.2	0.79

*mm* millimeter, *min* minutes

Of the cases with successful WEB deployment, additional stenting was more often used in the WEB 21 group (6/56, 10.7%) than in the WEB 17 group (1/88, 1.1%). This difference was significant in the unmatched series (p = 0.03), but lost significance after PS matching (p = 0.06).

**Fig. 2:**
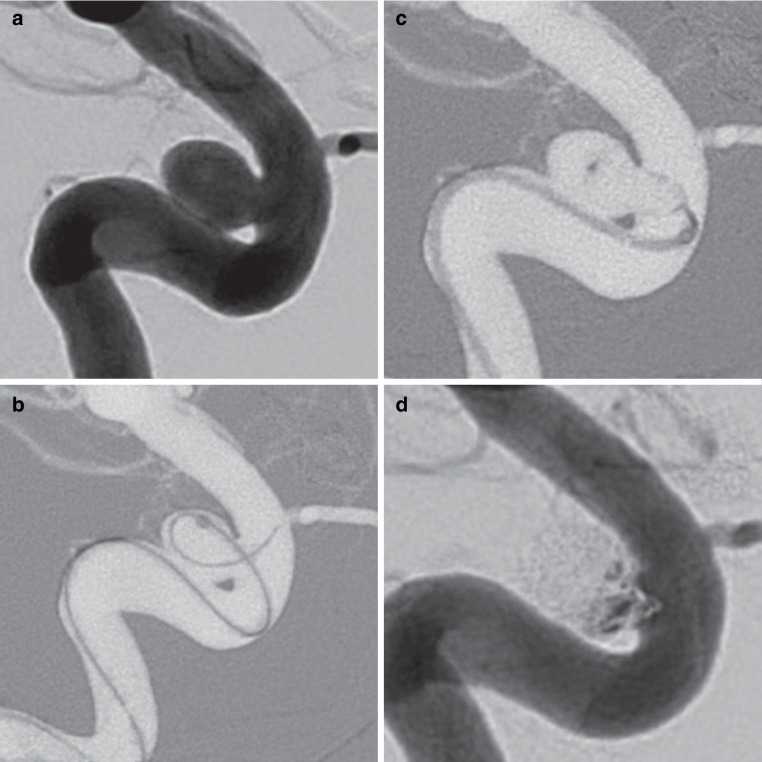
Digital subtraction angiography shows an unruptured aneurysm (width: 5.3 mm) in the concave wall of the paraophthalmic internal carotid artery (ICA) (**a**). The aneurysm was spherical in shape, making it suitable for treatment with a 6 mm WEB SLS. As a sidewall aneurysm, the angle between the aneurysm and the parent artery was approximately 90 degrees. Due to the right angled orientation and the curvy anatomy of the paraophthalmic ICA segment, it took several attempts to place the VIA 21 microcatheter inside the aneurysm, but a centered position of the tip was not possible (**b**). The WEB SLS 6 mm (WEB 21 system) was deployed, but after unfolding of the tip in the center part, the WEB could not be pushed into the aneurysm, and further deployment pushed the microcatheter back into the parent vessel (**c**). Therefore, the WEB had to be removed. The authors believe that treatment with the WEB 17 system would have been successful due to the lower profile and more flexible design and the availability of angled VIA microcatheters. The aneurysm was treated with balloon-assisted coiling in the same session, achieving near-complete occlusion (**d**)

**Fig. 3:**
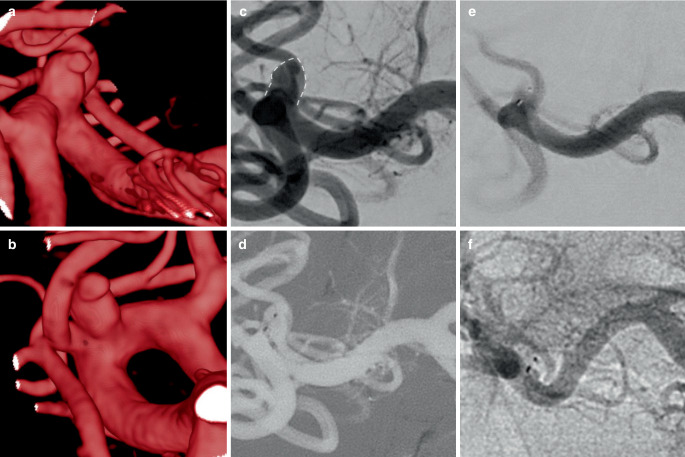
Three-dimensional reconstructions from rotational angiography show a wide middle cerebral artery bifurcation aneurysm (**a**, **b**). The width of the aneurysm is 3.1 mm and the height is 3.3 mm. The aneurysm is asymmetrically superiorly oriented at the bifurcation and has a bleb at the tip of the dome, making this aneurysm at high risk for spontaneous rupture. **c** shows the working projection, the dotted line highlights the shape of the aneurysm for better understanding. The aneurysm was probed with a 90° angled 0.017″ VIA microcatheter and a WEB 17 SL 4 × 2 mm was deployed (**d**). After detachment, the device seals the aneurysm neck and provides immediate contrast retention within the aneurysm (**e**). At the 1‑year follow-up, the unsubtracted DSA scan shows complete aneurysm occlusion (**f**)

The overall failure rate (deployment failure and adjunctive stent) was significantly higher with WEB 21 (10/60, 16.7%) than with WEB 17 (3/90, 3.3%, *p* < 0.01), which remained significant in the PS analysis (*p* = 0.03). An illustratory case of failed WEB 21 deployment, which might have succeeded with the WEB 17 system is shown in figure 2. Figure 3 shows a successful WEB 17 case. Overall, the WEB SL was used in 105 (70.0%) cases and the WEB SLS in 45 (30.0%), with no significant differences between the WEB 17 and 21 groups. The average width of the WEB 21 was 5.8 ± 1.1 mm compared to 5.5 ± 1.1 mm of the WEB 17, the difference was not significant (*p* = 0.09). The mean WEB/dome ratio was 1.14 ± 0.14 in the overall cohort with no significant differences between WEB 21 and 17. The mean fluoroscopy time was comparable for WEB 21 (24.6 ± 19.1 min) and WEB 17 (24.0 ± 18.8 min, *p* = 0.86) procedures.

### Complications and Clinical Outcome

Procedural complications are shown in Table 3. The overall major event rate was 1.3% (2/150) and the overall minor event rate was 2.0% (3/150). The combined major and minor neurological event rate was higher in the WEB 21 group (4/60, 6.7%) than in the WEB 17 group (1/90, 1.1%) without reaching statistical significance in the unmatched (*p* = 0.08) and the matched series (*p* = 0.36). The two major events were subarachnoid hemorrhage caused by aneurysm perforation with the distal tip of the WEB 21 in a middle cerebral artery (MCA) bifurcation aneurysm and perforation with the guide wire in a WEB 21 treated Acom aneurysm. The first patient died from subarachnoid hermorrhage, brain edema and multi-infarct syndrome, while the second patient was discharged to a rehabilitation center with a mRS 3. There was one additional aneurysm perforation caused by the tip of a WEB 21 during treatment of an Acom aneurysm with subarachnoid hemorrhage, but the patient had no neurological symptoms and was discharged with a mRS 0 after a short stay in the intensive care unit (minor event). There were no hemorrhagic complications with WEB 17 treatment.

**Table 3:** Procedural complications

Parameter	Unmatched series			Propensity score-matched WEB 17 cases	
	WEB 21 (*N* = 60)	WEB 17 (*N* = 90)	*P*	WEB 17 (*N* = 60)	*P*
**Neurologic events**	**4 (6.7%)**	**1 (1.1%)**	**0.08**	**1 (1.7%)**	**0.36**
*Minor*	*–*				
Stroke	1 (1.7%)	1 (1.1%)	–	1 (1.7%)	–
SAH	1 (1.7%)	0 (0%)		0 (0%)	
*Major*	*–*				
Stroke	0 (0%)	0 (0%)	–	0 (0%)	–
SAH	2 (3.3%)	0 (0%)		0 (0%)	
**Technical, asymptomatic complications**	**4 (6.7%)**	**4 (4.4%)**	**0.72**	**2 (3.3%)**	**0.68**
thromboembolic	4 (6.7%)	4 (4.4%)	–	2 (3.3%)	–
hemorrhagic	0 (0%)	0 (0%)		0 (0%)	
dissection	0 (0%)	0 (0%)		0 (0%)	

*SAH* subarachnoid hemorrhage

The other two minor events were minor strokes due to thromboembolic infarction caused by WEB 17 embolization of a posterior communicating artery (Pcom) aneurysm and WEB 21 embolization of an Acom aneurysm. Both patients had mild symptoms but fully recovered during the hospital stay (both mRS 0 at discharge). There were no major strokes in either group.

In addition to symptomatic complications, 4 (6.7%) asymptomatic thromboembolic complications occurred during WEB 21 treatment and 4 (4.4%, *p* = 0.72) during WEB 17 treatment. In 7 cases, the thrombus was dissolved with i.a. tirofiban infusion and in 1 case an additional stent was implanted to fix the thrombus to the vessel wall. There were no asymptomatic bleeding events and no arterial dissections.

Overall procedural morbidity was 1.3% (2/150) due to the two major hemorrhagic events in the WEB 21 group (3.3%), while there was no morbidity in the WEB 17 group (*p* = 0.16). There was no difference in morbidity rates after PS matching (*p* = 1.0).

### Angiographic Outcome

Angiographic results are shown in Table 4. WEB deployment failures were excluded from the angiographic outcome analysis. Short-term angiographic follow-up was available in 112 patients (46 WEB 21, 66 WEB 17). In the overall cohort, complete occlusion was achieved in 83 (74.1%) and adequate occlusion in 99 (88.4%). Comparable rates of complete and adequate occlusion were achieved with WEB 21 in 80.4 and 91.3%, respectively and with WEB 17 in 69.7 and 86.4%, respectively (*p* = 0.27).

**Table 4:** Angiographic outcome

Parameter	Unmatched series			Propensity score-matched WEB 17 cases	
	WEB 21	WEB 17	*P*	WEB 17	*P*
*Short-term FU (≤* *12 months)*	*N* *=* *46*	*N* *=* *66*	*–*	*N* *=* *43*	*–*
FU period	6.0 ± 2.5	6.4 ± 2.9	0.41	6.4 ± 2.8	0.13
Complete occlusion	37 (80.4%)	46 (69.7%)	0.27	28 (65.1%)	0.27
Neck remnant	5 (10.9%)	11 (16.7%)	–	8 (18.6%)	–
Aneurysm remnant	4 (8.7%)	9 (13.6%)		7 (16.3%)	
Retreatment	5 (10.9%)	2 (3.0%)	0.12	1 (2.3%)	0.20
*Mid-term FU (>* *12 months)*	*N* *=* *29*	*N* *=* *31*	*–*	*N* *=* *21*	*–*
FU period	24.7 ± 7.5	18.3 ± 5.1	< 0.01	23.0 ± 8.1	0.30
Complete occlusion	22 (75.9%)	20 (64.5%)	0.41	14 (66.7%)	0.42
Neck remnant	3 (10.3%)	6 (19.4%)	–	4 (19.0%)	–
Aneurysm remnant	4 (13.8%)	5 (16.1%)		3 (14.3%)	
Retreatment	0 (0%)	0 (0%)	1.0	0 (0%)	1.0

*FU* follow-up

Mid-term angiographic follow-up was available in 60 patients (29 WEB 21, 31 WEB 17). In the overall cohort, complete occlusion was achieved in 42 (70.0%) and adequate occlusion in 51 (85.0%). Comparable rates of complete and adequate occlusion were achieved with WEB 21 in 75.9 and 86.2%, respectively, and with WEB 17 in 64.5 and 83.9%, respectively (*p* = 0.41).

Seven (6.3%) aneurysms were retreated, 5 (8.5%) after WEB 21 treatment (2 Acom, 1 paraophthalmic ICA, 1 MCA bifurcation, 1 BA tip) and 2 (2.4%) after WEB 17 treatment (1 superior cerebellar artery, 1 paraophthalmic ICA). Retreatments consisted of stent-assisted coiling in 5 cases and flow diverter implantation in 2. All retreatments were performed within the 12-month FU period. Retreated aneurysms were excluded from further angiographic follow-up.

## Discussion

The present study demonstrates superior feasibility of the WEB 17, with an overall failure rate of 3.3% compared to 16.7% for WEB 21. The efficacy for both systems was comparable, with mid-term adequate occlusion rates of 86% for WEB 21 and 84% for WEB 17. Neurological complications occurred more often with WEB 21 treatment (6.7% vs. 1.1%), without reaching statistical significance, and the procedural morbidity rate was low with 3.3% for WEB 21 and 0% for WEB 17.

Comparative and well-matched studies are needed to scientifically evaluate the safety and efficacy of new devices. A prospective, randomized comparative study would be best, but this is not possible when the previous version of the device is no longer available. To date, three studies have compared the WEB 17 with its predecessor, the WEB 21. Pagano et al. compared 38 WEB 21 cases with 54 WEB 17 cases and König et al. compared 63 WEB 21 cases with 130 WEB 17 cases [6, 7]. In addition, we have described our initial experience with both devices in a previous publication [8]. However, the available studies are limited by small to moderate size and inhomogeneous patient cohorts. For example, ruptured aneurysms represent 7% in the Pagano et al. series and 26% in the König et al. series. Neither study made statistical adjustments for confounding baseline parameters or attempted retrospective matching. To address these limitations, the present study excluded ruptured aneurysms and used 1:1 propensity score matching, resulting in two comparable groups. In addition, the study included only the 4–7 mm device sizes, which were available for both systems and only the 3.0–6.5 mm aneurysm widths recommended by the manufacturer for these device sizes.

In the present study, WEB 17 had a lower failure rate, which may be related to the low-profile design of the WEB 17 system and the introduction of low-profile VIA 17 microcatheters. In our experience, both the VIA 17 and 21 microcatheters are very soft at the tip but provide reliable tip control, allow steam shaping and have good navigability into the aneurysm. However, the addition of 45 and 90° angled tips with the new VIA 17 makes it easier to catheterize aneurysms and deploy the WEB in angulated or sidewall aneurysms. In addition, the WEB 17 is more flexible than the WEB 21 by reducing the number of device wires. In contrast, the feasibility rates for both types were comparable in the studies by Pagano et al. (WEB 21: 97.4%, WEB 17: 94.4%) and König et al. (WEB 21: 95.2%, WEB 17: 98.5%) [6, 7]. As the WEB 21 was mainly implanted in the first half of the study period, increasing operator experience may have led to a better pre-selection for WEB implantation and consequently to lower failure rates, however fluoroscopy times—a surrogate parameter for operator experience—was comparable in both groups. In the prospective CLEVER study of the WEB 17, no procedural failures were reported in 163 cases, confirming the high feasibility of this device [4].

The combined major and minor event rate was higher in WEB 21 (6.7%) than in WEB 17 (1.1%), however, not reaching significance. In contrast, the complication rates of WEB 17 (13.7%) were slightly but not significantly higher than those of WEB 21 (5.4%) in the study by Pagano et al. [7]. König et al. reported comparable complication rates for WEB 21 (6.3%) and WEB 17 (6.2%). [6] The morbidity rate was low in the present series (1.5%) with no significant difference between WEB 21 and WEB 17. Similarly, there was no morbidity for both WEB 21 and WEB 17 in the Pagano study, while König et al. reported morbidity rates of 3.2% for WEB 21 and 2.5% for WEB 17.

These morbidity rates are consistent with benchmark WEB studies such as the 3 European studies WEBCAST, WEBCAST-2 and French Observatory (1.2%), WEB-IT (0.7%), and the WorldWideWEB Consortium (2.0%) [5, 9–11]. Likewise, the morbidity rate was 1.8% in the prospective CLEVER study on WEB 17 [4].

The WEB 17 system has fewer wires than the WEB 21, therefore it must be verified whether the WEB 17 provides similar efficacy in terms of aneurysm occlusion. It should be noted that the WEB 17 is available in half-step mm sizes for smaller aneurysms, which may favor occlusion rates through more adequate device sizing, however, these devices were not included in the present study. The increased learning curve may further favor the occlusion results of WEB 17. König et al. reported short-term (3 months) complete occlusion in 55.1% for WEB 21 and 65.5% for WEB 17 and adequate occlusion in 91.8% for WEB 21 and 86.9% for WEB 17 [6]. Pagano et al. reported mid-term (12 months) complete and adequate occlusion rates of 59.2 and 95.9% for the WEB 17 and 52.9 and 85.3% for the WEB 21 [7]. While both studies indicated improved efficacy with the WEB 17, these differences did not reach statistical significance in either study. In addition, the WEB 17 had lower complete occlusion rates than the WEB 21 in the present study, again without reaching statistical significance and with comparable adequate occlusion rates, which were within the range of the cited studies. Based on the available data, the WEB 17 appears to have at least similar safety and efficacy to the WEB 21 with potential advantages in device handling, warranting its continued clinical use.

### Limitations

This study has several limitations. The results are based on a retrospective analysis with a moderate number of patients. However, PS matching is utilized to create homogenous study cohorts. Treatment outcomes with WEB 17 may be favored by increased operator experience and better preselection of aneurysms. Short- and mid-term follow-up is incomplete and long-term angiographic follow-up information is lacking due to current unavailability. Finally, the lack of angiographic evaluation by a core laboratory raises the possibility of confounding the reported angiographic results.

## Conclusions

Our study shows improved feasibility of WEB 17 over WEB 21, with comparable treatment safety and efficacy. These results are confirmed by 1:1 propensity score matching, which strengthens the validity of these results. The improved feasibility may be partly related to an increasing learning curve and better aneurysm selection. In addition, the flexible design with the introduction of angled microcatheters may also improve procedural success. The available evidence on the WEB 17 justifies its further clinical use. Prospective multicenter studies are underway to define the long-term efficacy of this device.
